# Albanian Treatment for Ribbentrop's Chauffeur

**Published:** 1990-09

**Authors:** J. G. Dumoulin


**ALBANIAN TREATMENT FOR RIBBENTROP'S CHAUFFEUR**

Mortonhampstead has a famous hotel and golf course. Henry Cotton once described the short third hole there as one of the finest in the world; he must have visited it in early June when the rhododendrons are at their best.

Another pre-war visitor was Hitler's ambassador in London, Herr von Ribbentrop. He liked the place so much that he intended making it his country house after the war, so rumour had it.

I wonder if the German ambassador travelled down to Devon by car. The matter interests me because when I was a student at St Thomas's Hospital in 1939 there was considerable amusement and speculation when his chauffeur was admitted with "folies de grandeur". The diagnosis was obvious, and confirmed by a Wasserman Reaction test; he was suffering from general paralysis of the insane—GPI. What we modern doctors can only describe as ludicrous, was the treatment given to the mad chauffeur. Repeated rigors were induced by deliberately giving the wretched man malaria. When the consultant physician decided enough was enough, the malaria was treated with quinine sulphate.

Sometime later I learnt of the rationale for this useless treatment. In the First World War, a German Army doctor stationed in Albania had noted that in that country where syphilis was rife, the tertiary stage, whilst commonly marked by the presence of gummata, hardly ever appeared as tabes dorsalis or GPI. He also saw the vast amount of endemic malaria, particularly in the coastal regions. His conclusion was that malaria protected a syphilitic patient from acquiring neurological manifestations of the disease. One World War later, as a medical officer in SOE with the partisans in Albania, I was able to confirm his observations.

It may seem poetic justice to give a German treatment invented by a German, however illogically determined. Even teaching hospital consultants don't always get it right. Like the consultant obstetrician at the above mentioned hospital who showed us students a bloated stillborn baby and grossly oedematous placenta. "Typical case of syphilis" was his comment–in spite of a negative W.R.

Well in those days, the Rhesus Factor had not even been invented!

J. G. DUMOULIN

